# Oxidative stress induced by NOX2 contributes to neuropathic pain via plasma membrane translocation of PKCε in rat dorsal root ganglion neurons

**DOI:** 10.1186/s12974-021-02155-6

**Published:** 2021-05-06

**Authors:** Jing Xu, Shinan Wu, Junfei Wang, Jianmei Wang, Yi Yan, Mengye Zhu, Daying Zhang, Changyu Jiang, Tao Liu

**Affiliations:** 1 Center for Experimental Medicine, the First Affiliated Hospital of Nanchang University, Nanchang, 330006 Jiangxi China; 2 Department of Pediatrics, the First Affiliated Hospital of Nanchang University, Nanchang, 330006 Jiangxi China; 3 Department of Pain Medicine, the First Affiliated Hospital of Nanchang University, Nanchang, 330006 Jiangxi China; 4 Jisheng Han Academician Workstation for Pain Medicine, Huazhong University of Science and Technology Union Shenzhen Hospital, Shenzhen, 518052 Guangdong China

**Keywords:** Neuropathic pain, Dorsal root ganglion, NADPH oxidase 2, Reactive oxygen species, Protein kinase Cε

## Abstract

**Background:**

Nicotinamide adenine dinucleotide phosphate oxidase 2 (NOX2)-induced oxidative stress, including the production of reactive oxygen species (ROS) and hydrogen peroxide, plays a pivotal role in neuropathic pain. Although the activation and plasma membrane translocation of protein kinase C (PKC) isoforms in dorsal root ganglion (DRG) neurons have been implicated in multiple pain models, the interactions between NOX2-induced oxidative stress and PKC remain unknown.

**Methods:**

A spared nerve injury (SNI) model was established in adult male rats. Pharmacologic intervention and AAV-shRNA were applied locally to DRGs. Pain behavior was evaluated by Von Frey tests. Western blotting and immunohistochemistry were performed to examine the underlying mechanisms. The excitability of DRG neurons was recorded by whole-cell patch clamping.

**Results:**

SNI induced persistent NOX2 upregulation in DRGs for up to 2 weeks and increased the excitability of DRG neurons, and these effects were suppressed by local application of gp91-tat (a NOX2-blocking peptide) or NOX2-shRNA to DRGs. Of note, the SNI-induced upregulated expression of PKCε but not PKC was decreased by gp91-tat in DRGs. Mechanical allodynia and DRG excitability were increased by ψεRACK (a PKCε activator) and reduced by εV1-2 (a PKCε-specific inhibitor). Importantly, εV1-2 failed to inhibit SNI-induced NOX2 upregulation. Moreover, the SNI-induced increase in PKCε protein expression in both the plasma membrane and cytosol in DRGs was attenuated by gp91-tat pretreatment, and the enhanced translocation of PKCε was recapitulated by H_2_O_2_ administration. SNI-induced upregulation of PKCε was blunted by phenyl-*N*-tert-butylnitrone (PBN, an ROS scavenger) and the hydrogen peroxide catalyst catalase. Furthermore, εV1-2 attenuated the mechanical allodynia induced by H_2_O_2_

**Conclusions:**

NOX2-induced oxidative stress promotes the sensitization of DRGs and persistent pain by increasing the plasma membrane translocation of PKCε.

**Supplementary Information:**

The online version contains supplementary material available at 10.1186/s12974-021-02155-6.

## Background

Neuropathic pain is one of the most intractable neurological diseases due to the lack of effective clinical treatments to date [1]. Oxidative stress generated by superoxide anion (·O_2_^−^), hydroxyl radical (·OH), and hydrogen peroxide (H_2_O_2_) plays a crucial role in multiple pathological pain states, such as neuropathic pain [2, 3], migraine-related pain [4], and inflammatory pain [5]. A proposal that O_2_^−^ was intimately involved in the inflammatory response was raised as early as the 1970s [6]. It implicates that reactive oxygen species (ROS) is one of mediators of neuroinflammation [7] as·O_2_^−^, H_2_O_2_, and·OH occurs at the site of inflammation [8]. Accordingly, scavenging reactive oxygen species (ROS) with phenyl-*N*-tert-butylnitrone (PBN) or catalyzing H_2_O_2_ with catalase in the spinal dorsal horn (SDH) has been shown to alleviate nerve injury-induced mechanical allodynia or antinociceptive tolerance to morphine [2, 9]. Nicotinamide adenine dinucleotide phosphate (NADPH) oxidase (NOX) is one of the major sources of ROS in the nervous system and includes seven members: NOX1-5 and Duox1-2 [10]. Our recent study demonstrated that nicotinamide adenine dinucleotide phosphate oxidase 2 (NOX2)-induced ROS production participates in the development of persistent pain through increasing the excitability of SDH neurons of rats under a high-frequency stimulation (HFS) at the sciatic nerve [11]. Moreover, Kallenborn-Gerhardt et al. showed that nerve injury activated NOX2-mediated oxidative stress in dorsal root ganglia (DRGs), leading to neuropathic pain behavior, which were reduced in Nox2-deficient mice [12]. This is suggested that NOX2 plays a critical role in neuropathic pain. However, whether NOX2-ROS could promote neuropathic pain though regulating the peripheral sensitization in DRGs is still unclear. =

In addition to oxidative stress, the activation of protein kinase C (PKC) in DRGs plays a crucial role in pathological pain [13–15]. The specific distribution of PKC via plasma membrane translocation is a marker of PKC activation. In small, medium, and large DRG neurons, plasma membrane translocation of PKC has been shown to produce chemotherapy-induced peripheral neuropathy [16] or bone cancer-induced hyperalgesia [17]. Moreover, ROS production by NOX2 can modulate PKC activity, resulting in pain hypersensitivity in the SDH [18]. However, whether NOX2-induced oxidative stress in rodent DRGs after nerve injury can promote plasma membrane translocation of PKC remains unknown.

In this study, we investigated whether oxidative stress induced by NOX2 in DRGs leads to mechanical allodynia in spared nerve injury (SNI) models of neuropathic pain and the underlying mechanisms. We found that NOX2-mediated ROS and H_2_O_2_ activation induced hyperexcitability of DRG neurons in SNI rats. In addition, this activation promoted PKCε activation and plasma membrane translocation, which led to mechanical allodynia. In summary, activation of NOX2-ROS-PKCε signaling in DRGs was necessary and sufficient to generate neuropathic pain.

## Methods

### Animals and surgery

Male Sprague-Dawley (SD) rats weighting 200–250 g were used unless otherwise mentioned. They were obtained from the Animal Center of Nanchang University. The rats were housed in a temperature-controlled room, maintaining at 24 ± 1 °C and 50–60% humidity, with a 12:12-h light/dark cycle. Food and water were available ad libitum. Experimental procedures were approved by Institutional Animal Care and Use Committee of Nanchang University and according to ARRIVE guidelines [19]. Spared nerve injury (SNI) was performed as previously described [20]. Briefly, under isoflurane (1.5–2.5%) anesthesia, the sciatic nerve of the left hindlimb was exposed to common peroneal, tibial, and sural nerves, and then the former two nerves were ligated and sectioned, whereas the sural nerve was kept intact. In the sham group, the sciatic nerve was only exposed without ligation or cut. All of the animals were assigned to different treatment groups randomly.

### Acute local application of drugs onto DRG in vivo

Local application of drugs onto DRGs was done as previously described [21] with slight modifications. Briefly, after rats were anesthetized by isoflurane (1.5–2.5%), a midline incision was made at the L4–L6 spinal level, muscles were gently pulled aside to visualize the caudal edge of L4–L6 vertebra. A small, partial laminectomy by rongeur (501269, WPI, Florida USA) was used to expose the left L4–L6 DRGs. Drugs were dissolved in 2 μl normal saline by gelatin sponge. The incision was closed with sutures, and rats with hind limb paralysis or paresis after surgery were excluded. The drugs used were NOX2-specific blocking peptide gp91-tat (AS-63818, Anaspec, Fremont, CA), PKCε-specific blocking peptide εV1-2 (AS-62187, Anaspec), PKCε-specific activator peptide ψεRACK (AS-63818, Anaspec), H_2_O_2_ (**323381**, Sigma, St. Louis, MO), ROS scavenger PBN (B7263, Sigma), and catalase (C1345, Sigma).

To verify if the treatment of drug was limited to the specific DRG, CFSE (21888, Sigma; 100 μM in 2 μl each DRG), a fluorescent dye, was applied onto L5 DRG by gelatin sponge. One hour after application, the animal was sacrificed, and L5 DRG and proximal spinal cord segment were excised. The DRG sections (16 μm) and spinal cord sections (25 μm) were cut in a cryostat (CM1950, Leica, Nussloch, Germany). Fluorescent images were obtained with a laser confocal microscope (LSM700, Zeiss, Germany).

### AAV-shRNA preparation and transfection

For adeno-associated virus 9 (AAV9) construction, the shRNA for NOX2 and scramble shRNA were cloned into the pAKD-CMV-bGlobin-mCherry-H1-shRNA (cybb) (Obio Technology Corp., Ltd. Shanghai, China). The sequences of cybb shRNA and scramble shRNA are as follows:

cybb shRNA: 5-′GTCATCACACTGTGTCTTA-3′

scramble shRNA: 5-′TTCTCCGAACGTGTCACGT-3′

Injection of AAV vectors into DRGs was performed according to a previous report [22] with slight modification. Briefly, after anesthesia with isoflurane (1.5–2.5%), a midline incision was made at the L4–L6 spinal level. Partial laminectomy was carried out to expose the left L4–L6 DRGs. A glass micropipette (tip diameter 10–20 μm) filled with AAV stock connecting to a microsyringe (KD Scientific, MA, USA) was attached to a manipulator. One microliter of AAV-cybb-shRNA or AAV-scramble-shRNA stock (titer matched to 1 × 10^12^ IU/ml) was injected (1 μl/10 min) into DRGs with a depth of 0.5 mm. After 21 days of shRNA transfection, the rats received SNI surgery and behavior tests.

### Assessment of mechanical sensitivity

Mechanical sensitivity of rats were assessed with the up–down method following the previous study [23]. After acclimation for 30 min on a plastic box, a set of Von Frey filaments (North coast, USA) with logarithmically incremental stiffness from 0.4 to 15.0 g were applied alternately to the plantar surface of the hind paw. Each stimulus consisted of a maximum of 6 s application of the filament, quick withdrawal or licking on the paw in response to the stimulus was considered a positive response. The behavioral test was performed on double-blinded design.

### Isolation and culture of DRG neurons

DRG neurons from SD rats (60–80 g) were dissociated as previously described [24]. Briefly, L4–L6 DRGs were excised in ice-cold DMEM/F12 medium (10565018, GIBCO, USA) and mechanically dissociated. After digested with tyrisin (T9201, Sigma, USA) and collagenase (C9891, Sigma, USA) for 30 min in 37 °C, DRG neurons were seeded onto cover slips coated with poly-l-lysine (P7890, Sigma, USA) in a humidified atmosphere (5% CO_2_, 37 °C) for up to 4 h and then were used for patch-clamp recording.

### Whole-cell patch-clamp recording

Whole-cell patch-clamp recording was randomly selected from small size (< 25 μm) and medium size (25–35 μm) DRG neurons, and was obtained by an EPC-10 amplifier and the PatchMaster program (HEKA Electronics, Lambrecht, Germany) as previously described [25]. The membrane capacitance was read from the amplifier by PatchMaster. Patch pipettes with 3–5 MΩ resistance were pulled from borosilicate glass (World Precision Instruments, Sarasota, FL, USA) using a Sutter P-97 puller (Sutter Instruments, Novato, CA). For current-clamp recording, the extracellular solution contained (in mM) 117 NaCl, 3.6 KCl, 1.2 NaH_2_PO_4_·2H_2_O, 2.5 CaCl_2_·2H_2_O, 1.2 MgCl_2_·6H_2_O, 25 NaHCO_3_, 11 d-glucose, and 2 Sodium pyruvate (pH = 7.4 adjusted with NaOH, 300 mOsm). The pipette solution contained (in mM) 130 K-gluconate, 5 KCl, 10 Na_2_-Phosphocreatine, 0.5 EGTA, 10 HEPES, 4 Mg-ATP, and 0.3 Li-GTP. (pH = 7.3 adjusted with KOH, 295 mOsm). The action potentials (APs) of DRG neurons were elicited by a series of depolarizing currents every 10 s with step intervals of 10 pA from 0 to 400 pA over a period of 500 ms to measure the current threshold (rheobase). The current that induced the first AP was defined as 1×rheobase. The peak potential and half-width of APs were analyzed by Clampfit software (pClamp10, Molecular Devices). The neuronal input resistance was determined by measuring the voltage response to a depolarizing current (10 pA, 500 ms) from RMP in current-clamp mode.

### Western blotting

The L4–L6 DRGs were dissected in cold RIPA buffer. Plasma membrane protein and cytosol protein were isolated with the protein extraction kit (SM-005, Invent Biotechnologies, MN, USA) following the manufacturer’s instructions. The protein samples were separated and transferred onto a polyvinylidene fluoride (PVDF) membrane (Bio-Rad Laboratories, Inc., CA, USA). The PVDF membranes were incubated in blocking buffer for 1 h at room temperature followed by incubating in a primary antibody against NOX2 (1:1000, ab80508, Abcam, Bristol, UK), PKC (1:1000, ab31, Abcam), PKCε (1:1000, ab63638, Abcam), transferrin receptor (TfR) (1:1000, QG215340, Invitrogen, USA), and β-actin (1:1000, 20536, Proteintech, IL, USA) overnight at 4 °C, and then the membranes were incubated in HRP-conjugated secondary antibody. Imaging system (iBright FL1000, Thermo Fisher Scientific, MA, USA) was used to detect the immunocomplexes by chemiluminescence (ECL) solution (Millipore Bioscience Research Reagents, USA). The immunostained bands were quantified by ImageJ software (Version: k 1.45).

### Immunocytochemistry and immunohistochemistry

DRG neurons on coverslips were fixed by 4% paraformaldehyde (PFA) for 10 min at room temperature. After blocking with 10% donkey serum and 0.3% Triton X-100 for 1 h, the neurons were incubated with rabbit anti-PKCε (1:200, ab63638, Abcam) overnight at 4 °C. The cultured neurons were then incubated with FITC secondary antibody (1:200, Jackson ImmunoResearch, West Grove, PA) for 1 h at room temperature.

For frozen section, rats were transcardially perfused with 4% PFA. The L4–L6 DRGs were dissected and postfixed in 4% PFA for 1 h and dehydrated in 30% sucrose for over 3 nights. The DRG sections (16 μm) were cut in a cryostat (CM1950, Leica, Nussloch, Germany) and then incubated with primary antibodies against NOX2 (1:100, DF6520, Affinity Biosciences, China), PKCε (1:200, ab63638, Abcam), or 8-OHG (1:200, ab10802, Abcam) were incubated together with antibody for NF200 (1:200, N0142, Sigma), CGRP (1:200, ab81887, Abcam), IB4 (1:50, L2895, Sigma), CD11b (1:200, CBL1512, Sigma), or glial fibrillary acidic protein (GFAP, 1:500, Cell Signaling Technology), followed by cy3-conjugated (1:400, Jackson ImmunoResearch) or FITC secondary antibodies (1:200, Jackson ImmunoResearch).

Fluorescent images of DRG neurons on coverslips or DRGs sections were obtained with the laser confocal microscope (LSM700, Zeiss, Germany). Determination of the PKCε membrane translocation was based on the fluorescence intensity of the cells as previous reports [5, 16]. Briefly, the fluorescence intensity along a straight line across the neuronal soma was measured by Zen software (Zeiss, Germany). The ratio of averaged fluorescence intensity of the membrane region against the total fluorescence intensity of neuronal soma was demonstrated. The size of cultured DRG neurons was classified as small: < 600 μm^2^, medium: 600–1200 μm^2^, and large: > 1200 μm^2^ [16]. For quantification of immunofluorescence staining, 8-OHG-immunoreactivity (IR) in L4–L6 DRGs per section was measured by ImageJ (Version: k 1.45).

### Measurement of hydroxyl radical (·OH)

Level of ·OH was analyzed by Cell Hydroxyl Radical Colorimetrical Assay Kit (GMS10124.4, GenMed Scientifics Inc., MA, USA) according to the manufacturer’s instructions. Briefly, DRGs tissue in 400 ml medium was collected in a 5 ml glass tube, and reacted with reaction mixtures provided by the kit at room temperature for 30 min. Absorbance at a wavelength of 420 nm was measured by a spectrophotometer (U-2900, HITACHI, Japan).

### Statistics analysis

Data were shown as means ± SEM, and were analyzed by GraphPad Prism 8.4 software. The sample sizes were based on our previous research. The Shapiro-Wilk test was used to check the normality of data, which were considered normally distributed when *p* > 0.05; otherwise, non-parametric tests were used. Homogeneity of variance was be verified by Brown–Forsythe tests, when *p* < 0.05, data was analyzed by one-way ANOVA followed by Dunnett’s T3 multiple comparisons test. Two-tailed unpaired Student’s *t* test was used for analyses with two groups; one-way ANOVA followed by Tukey’s post hoc test was used to compare multiple groups. Nonparametric analysis was used when datasets did not pass the normality test. Kruskal-Wallis or Friedman test was used for multiple group statistical evaluation followed by Dunn’s multiple comparisons test. The Mann-Whitney test was used for analyses with two groups. *p* < 0.05 was considered statistically significant.

## Results

### SNI surgery induces persistent upregulation of NOX2 in DRGs

**Fig. 1.**
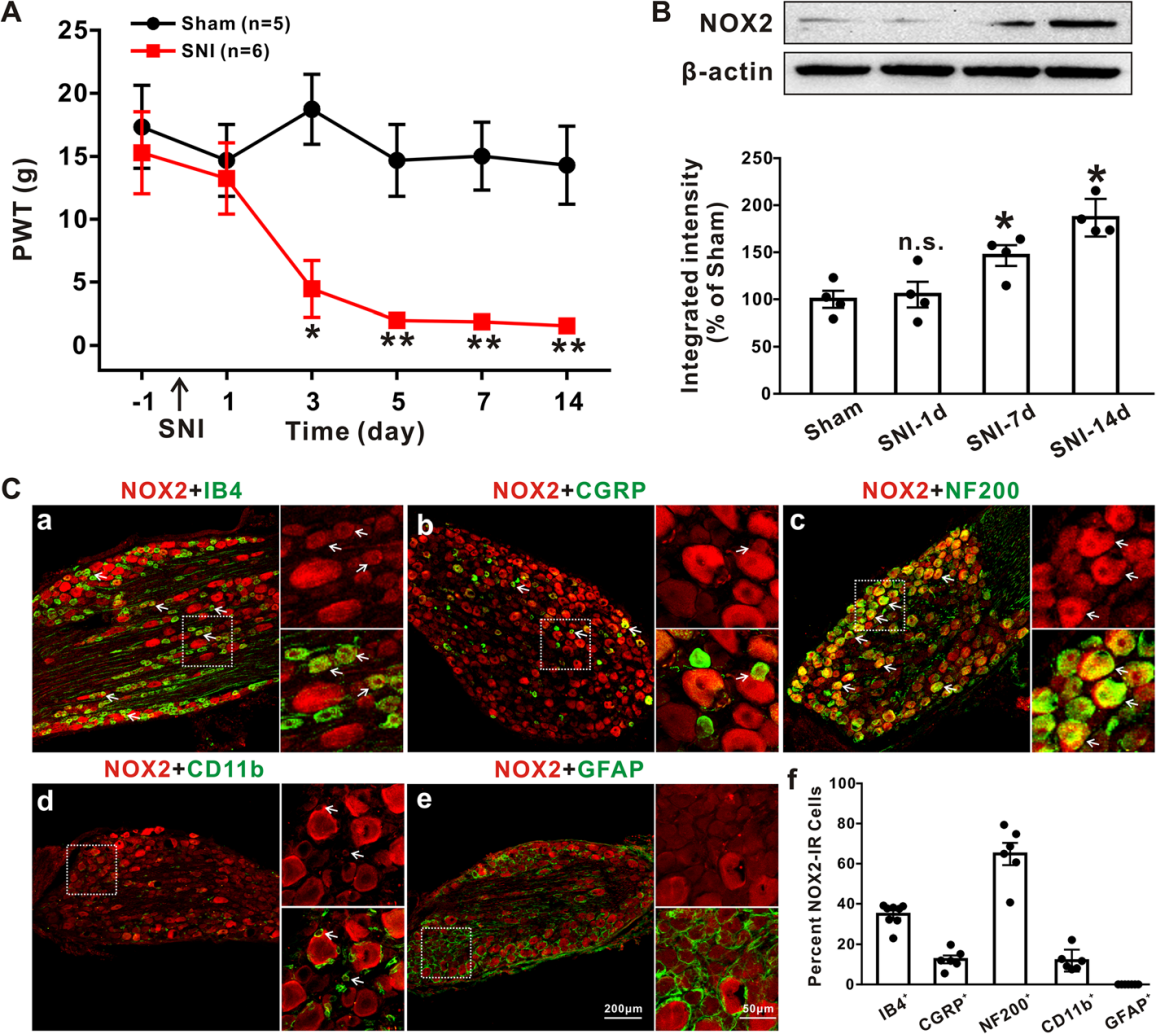
SNI induces NOX2 upregulation in DRG neurons. **a P**aw withdrawal threshold (PWT) after SNI (*n* = 5–6/group). Mann–Whitney *U* test. *p < 0.05, **p < 0.01 versus the sham group. **b** NOX2 expression in L4–L6 DRGs from rats in (**a**) at 1, 7, and 14 days after SNI and sham surgery was determined by Western blotting (*n* = 4/group). n.s. indicates not significant. One-way ANOVA followed by Tukey’s test. *p < 0.05 versus the sham group. **c** Representative double-immunofluorescence staining showing the colocalization of NOX2 with IB4 (**c**(a)), CGRP (**c**(b)), NF-200 (**c**(c)), and CD11b (**c**(d)) but not GFAP (**c**(e)) at day 7 after SNI. **c**(f) The histogram shows the percentage of NOX2-positive cells among the various cell markers (*n* = 6/group)

The 50% paw withdrawal threshold (PWT) in the ipsilateral hind paw was significantly decreased at day 3 (*p* < 0.05) and persisted until day 14 (*p* < 0.01) after SNI surgery (Fig. 1a, compared with the sham-operation group, Mann–Whitney *U* test). To investigate the expression of NOX2 in DRGs, we next performed Western blot analysis of NOX2 at 1, 7, and 14 days after SNI. Compared with the sham operation, SNI significantly increased NOX2 protein expression from 100 ± 6.83 to 146.62 ± 8.35% at day 7 and 186.84 ± 7.55% at day 14 (Fig. 1b, *p* < 0.05, one-way ANOVA followed by Tukey’s test). Double immunofluorescence staining showed that NOX2 colocalized with IB4-labeled nonpeptidergic neurons (34.75 ± 1.96%, Fig. 1c(a)), CGRP-labeled peptidergic neurons (12.47 ± 1.95%, Fig. 1c(b)), NF-200-labeled neurons (64.85 ± 5.56%, Fig. 1c(c)), and CD11b-labeled macrophages (11.89 ± 2.22%, Fig. 1c(d)) but not GFAP-labeled satellite glial cells (Fig. 1c(e)) in the L4–L6 DRGs of rats at day 7 after SNI. The percentages of the five markers that expressed NOX2-IR in the DRGs are shown in Fig. 1c(f). Similar results were observed in the sham group, except that NOX2 did not colocalize with CD11b **(**Fig. S1). These findings suggest that SNI induces a long-lasting increase of NOX2 in DRGs, which is mainly expressed in neurons but not glia.

### Local application of NOX2-blocking peptides or shRNA attenuates SNI-induced mechanical allodynia and hyperexcitability of DRG neurons

**Fig. 2:**
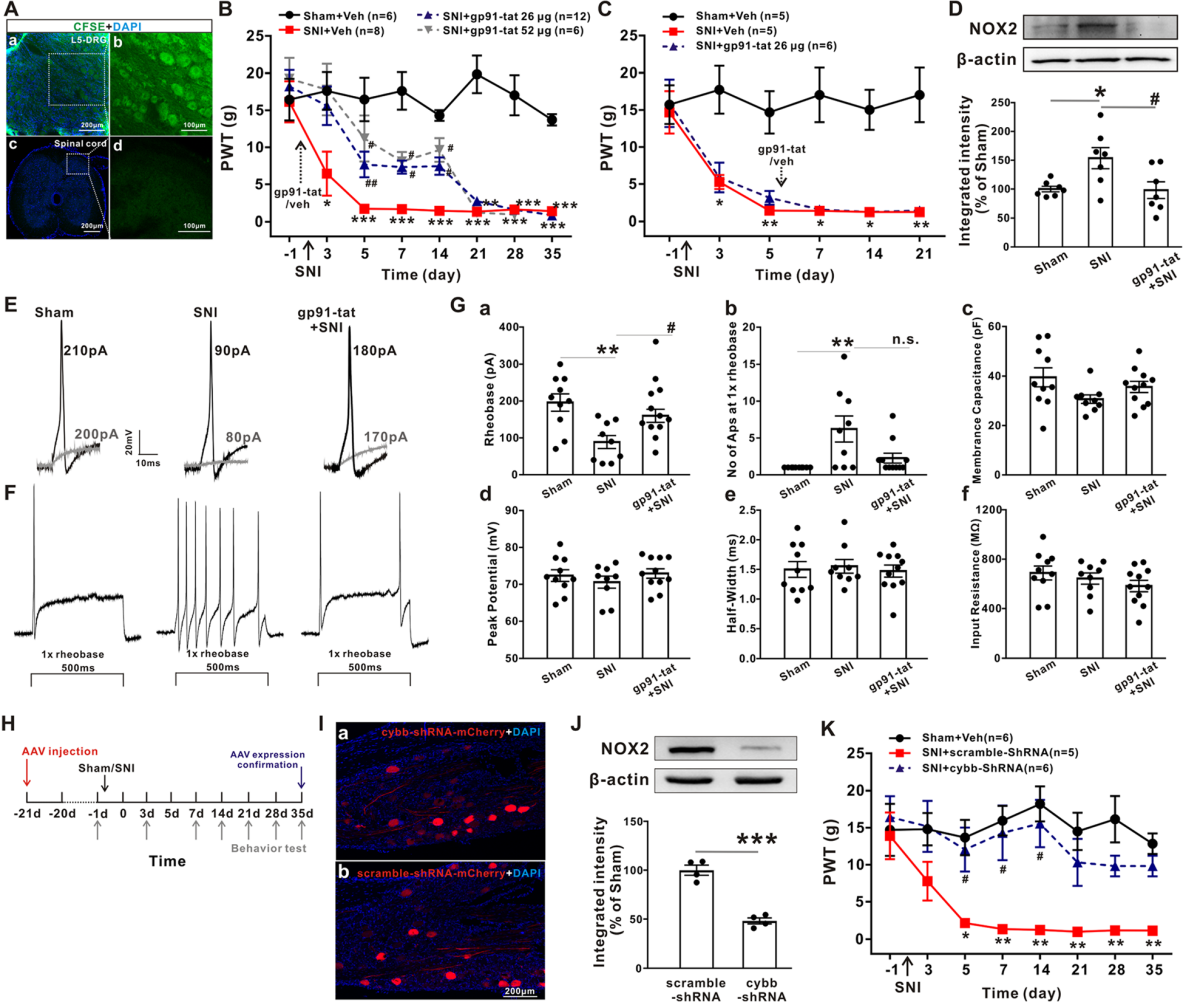
Pretreatment with NOX2-blocking peptides or shRNA attenuates SNI-induced mechanical allodynia and hyperexcitability in DRG neurons. **a** Images of the L5 DRG (**a**(a, b)) and proximal spinal cord (**a**(c, d)) after the local application of CFSE (100 μM in 2 μl) by gelatin sponge. **b**, **c** Local application of gp91-tat (26 μg or 52 μg) to L4-L6 DRGs before (**b**) but not after (**c**) SNI surgery significantly attenuated mechanical allodynia at 3–14 days after SNI. The dotted arrow indicates the time of gp91-tat or vehicle application. Kruskal-Wallis test followed by Dunn’s multiple comparison test. *p < 0.05, **p < 0.01, ***p < 0.001 versus the sham group; #*p* < 0.05, ##*p* < 0.01 versus the vehicle-treated SNI group. **d** Western blotting showed that pretreatment with gp91-tat decreased NOX2 expression in L4-L6 DRGs at 7 days after SNI (*n* = 7). One-way ANOVA followed by Tukey’s test. *p < 0.05 versus the sham group, #*p* < 0.05 versus SNI group. **e** Representative traces illustrating the action potentials and their rheobase currents recorded in L4–L6 DRG neurons. **f** Representative traces showing the action potentials elicited by 1×rheobase in L4-L6 DRG neurons. **g** Quantification analysis of rheobase currents (a), one-way ANOVA followed by Tukey’s test. **p < 0.01 versus the sham group, #*p* < 0.05 versus the SNI group. Numbers of action potentials (b), Kruskal-Wallis test followed by Dunn’s multiple comparison test. **p < 0.01 versus the sham group. Membrane capacitances (c). Peak amplitudes (d) and half-widths (e) of action potentials, as well as input resistance (f) in each group (*n* = 9–11/group). **h** Experimental diagram showing the timeline of AAV-shRNA injection and behavioral tests. **i**(a-b) Representative immunofluorescence staining showing the expression of cybb-shRNA-mCherry or scramble-shRNA-mCherry in transduced cells. **j** Western blotting analysis showed that cybb-shRNA injected into the DRGs diminished the protein expression of NOX2 (*n* = 4). Two-tailed unpaired Student’s *t* test. ***p < 0.001 versus scrambled shRNA. **k** NOX2 cybb-shRNA but not scrambled shRNA significantly abolished the initiation of mechanical allodynia after SNI (*n* = 5–6/group). Kruskal-Wallis test followed by Dunn’s multiple comparison test. *p < 0.05, **p < 0.01 versus the sham group; #*p* < 0.05 versus the cybb-shRNA group

It has been reported that gp91-tat, a 20-amino acid NOX2-blocking peptide that prevents the binding of p47 phox to gp91 phox and blocks enzyme assembly and activation [26]. To examine whether increased NOX2 expression in DRGs was responsible for SNI-induced pain hypersensitivity, we locally applied gp91-tat to ipsilateral L4–L6 DRGs 2 hours before SNI surgery. Site-specific drug delivery was verified by CFSE staining, as the CFSE signal was confined to the L5 DRG (Fig. 2a(a) and (b)) but not the proximal spinal cord (Fig. 2a(c) and (d)). Figure 2b shows that gp91-tat pretreatment significantly decreased the 50% PWT at day 3, and the effect persisted until day 14 after SNI. In contrast, the 50% PWT did not markedly differ between vehicle- and gp91-tat-treated SNI rats when applied 5 days after surgery (Fig. 2c). Consistently, Western blot analysis revealed that gp91-tat pretreatment reversed the SNI-induced upregulation of NOX2 in DRGs at day 7 after surgery (Fig. 2b).

Because the hyperexcitability of DRG neurons is critical for neuropathic pain development following peripheral nerve injury [27], we next tested whether the attenuation of SNI-induced mechanical allodynia by the NOX2-blocking peptide gp91-tat occurred by decreasing the hyperexcitability of DRG neurons. Whole-cell patch clamp recordings were obtained from small (< 25 μm) and medium (25–35 μm) L4–L6 DRG neurons to examine the characteristics of the APs of DRG neurons. To do this, DRG neurons were prepared from sham rats, rats at 7 days after SNI, and gp91-tat-pretreated rats at 7 days after SNI. As shown in Fig. 2e and g(a), the rheobase was significantly decreased in SNI rats at 7 days after surgery (88.89 ± 17.75 pA) compared with that of sham rats (196.00 ± 23.49 pA, *p* < 0.01, one-way ANOVA followed by Tukey’s test). However, compared with SNI rats, pretreatment with gp91-tat induced a profound increase in the rheobase (160 ± 17.6 pA, *p* < 0.05, one-way ANOVA followed by Tukey’s test). Next, we examined the number of APs elicited by 1× rheobase, which was increased by SNI to 6.22 ± 1.77 (Fig. 2f and g(b), *p* < 0.05, Kruskal-Wallis test followed by Dunn’s multiple comparison test). Pretreatment with gp91-tat decreased the number of APs (1.82 ± 0.40, *p* = 0.3411, Kruskal-Wallis test followed by Dunn’s multiple comparison test) but did not reach statistical significance, indicating a trend toward inhibiting DRG overexcitation following SNI. The membrane capacitance, peak potential, half width of APs, and input resistance were not different among the three groups (Fig. 2g(c, d)). Taken together, these results confirm that NOX2 activation in DRG neurons plays an essential role in SNI-induced neuropathic pain by increasing DRG excitability.

To further confirm the effect of NOX2 on neuropathic pain, we used a cybb-shRNA to knockdown the expression of NOX2. AAV-cybb-shRNA-mCherry or AAV-scramble-shRNA-mCherry was injected into the DRGs of the operation side 21 days before SNI (Fig. 2h). The immunohistochemical results demonstrated that cybb-shRNA-mCherry and scramble-shRNA-mCherry were expressed in DRGs at day 35 after SNI (Fig. 2i). Western blot further showed that the expression of NOX2 was downregulated after cybb-shRNA compared to scramble shRNA application (Fig. 2j). Behavioral tests also demonstrated that cybb-shRNA induced a marked reduction in the 50% PWT at 5, 7, and 14 days after SNI (Fig. 2k).

### Oxidative stress induces mechanical allodynia and hyperexcitability of DRG neurons

**Fig. 3:**
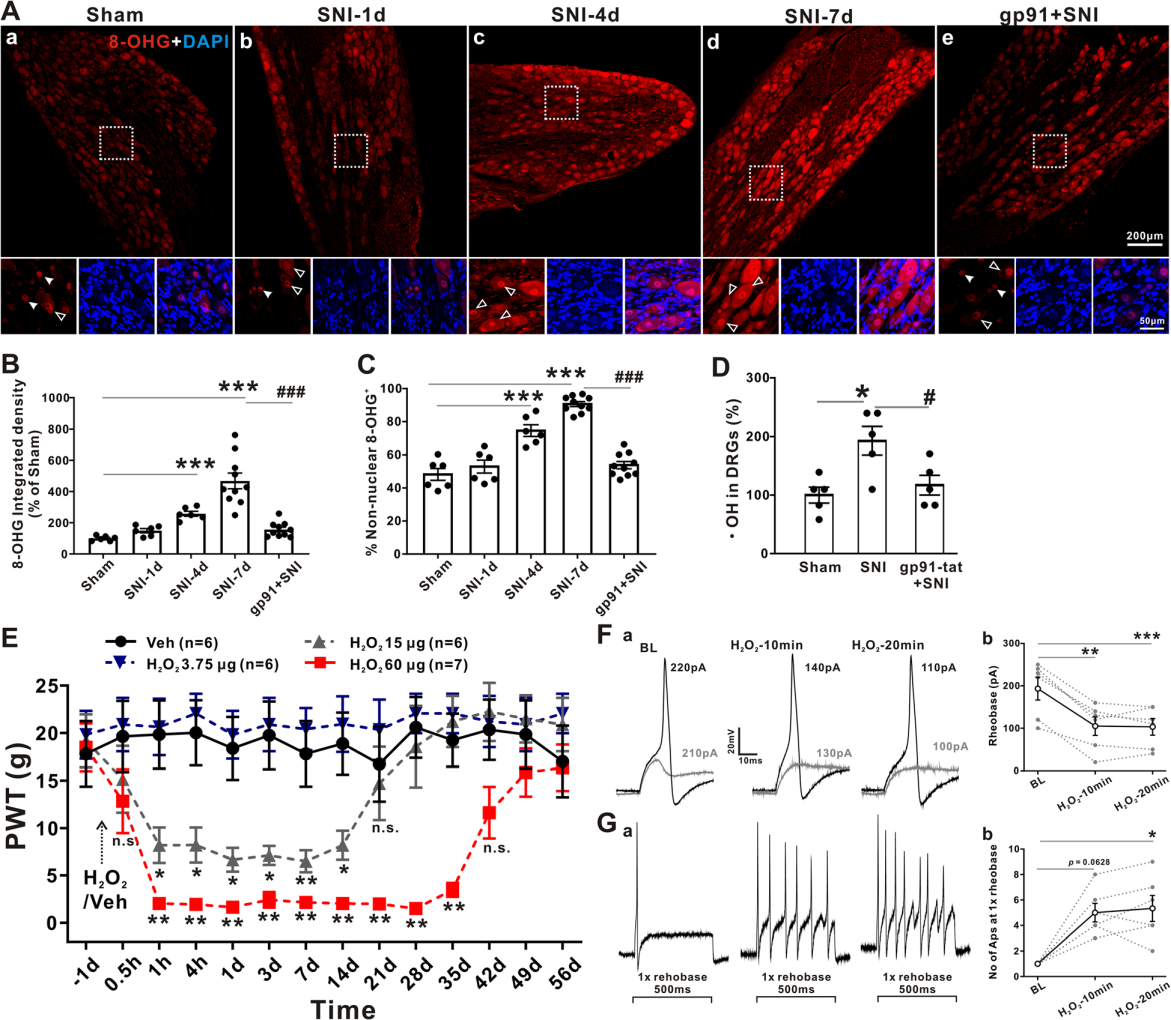
Oxidative stress generated by NOX2 induces mechanical allodynia and hyperexcitability in DRG neurons. **a(**a–e) Representative staining of 8-OHG in DRGs at 1, 4, and 7 days after SNI, as well as in gp91-tat-pretreated DRGs at 7 days after SNI. Open arrowheads show nonnuclear 8-OHG–positive neurons, and filled arrowheads show nuclear 8-OHG–positive neurons. **b** Quantification of the 8-OHG-IR intensity (*n* = 6–10/group). One-way ANOVA followed by Dunnett’s T3 multiple comparisons test. ***p < 0.001 versus the sham group; ###*p* < 0.001 versus the SNI group. **c** Nonnuclear 8-OHG–positive neurons as a percentage of total neurons in DRGs (*n* = 6–10/group). One-way ANOVA followed by Tukey’s test. ***p < 0.001 versus the sham group; ###*p* < 0.001 versus the SNI group. **d** Gp91-tat pretreatment inhibited SNI-induced ·OH generation (*n* = 5/group). One-way ANOVA followed by Tukey’s test. *p < 0.05, versus the sham group; #*p* < 0.05 versus the SNI group. **e** Time-related effects of 3.75, 15, and 60 μg of H_2_O_2_ on the 50% PWT. The dotted arrow indicates the time of H_2_O_2_ or vehicle application (*n* = 6–7/group). Mann–Whitney *U* test. *p < 0.05, **p < 0.01 versus the vehicle group. **f** Representative traces of rheobase currents (*n* = 6). One-way ANOVA followed by Tukey’s test. **p < 0.01, ***p < 0.001 versus baseline (BL). **g** Currents elicited by 1× rheobase before and at 10 min and 20 min after the bath application of H_2_O_2_ (*n* = 6). Friedman test followed by Dunn’s multiple comparison test. *p < 0.05 versus BL

It has been well documented that oxidative stress caused by ROS (e.g., superoxide and ·OH) and H_2_O_2_, which may be generated by NOX, is crucial in the development and maintenance of neuropathic pain in the central and peripheral nervous systems [3, 28, 29]. To investigate the role of SNI-induced oxidative stress in DRGs, we used 8-OHG-IR to examine oxidized nucleic acids produced by cellular ROS damage. We found that the levels of 8-OHG-IR in the DRGs were increased at days 4 and 7 after SNI, whereas pretreatment with gp91-tat reversed the increase in 8-OHG-IR at day 7 after SNI (Fig. 3a and b). Figure 3c shows that nonnuclear 8-OHG immunostaining significantly increased at day 4 after SNI, whereas pretreatment with gp91-tat reversed this increase. Nonnuclear 8-OHG immunostaining indicates oxidative stress in mitochondria [30], which produce superoxide that is converted to H_2_O_2_ by superoxide dismutase (SOD) [7]. Double immunostaining demonstrated that 8-OHG colocalized with IB4-, CGRP-, and NF-200-positive DRG neurons but not GFAP-positive glial cells at 7 days after SNI (Fig. S2A). The level of ·OH, which is the protonated form of ·O_2_^−^, was increased in the DRG tissue of the SNI group. This increase at 7 days after SNI was blocked by pretreatment with gp91-tat (Fig. 3d).

Next, we investigated the effect of the direct application of H_2_O_2_ to DRGs on pain behavior in naïve rats. Notably, the 50% PWT dose-dependently showed a rapid (1 h) and persistent (14 to 35 days) decrease after the local application of H_2_O_2_ (Fig. 3e). Accordingly, the level of ·OH was increased at 4 days after the administration of 60 μg of H_2_O_2_ (Fig. S2B). However, Western blotting showed that the same dose of H_2_O_2_ had no effect on the protein expression of NOX2 (Fig. S2C).

To explore the cellular mechanism underlying the rapid effect of H_2_O_2_ on mechanical allodynia, we next examined whether H_2_O_2_ could modulate the excitability of DRG neurons. As shown in Fig. 3f and g, compared to the sham control (193.33 ± 26.79 pA), the 10-min bath application of 500 μM H_2_O_2_ decreased the rheobase (105.00 ± 21.87 pA) (*p* < 0.01, one-way ANOVA followed by Tukey’s test) and increased the number of APs in response to the 1× rheobase (5.00 ± 0.73, *p* = 0.0628, Friedman test followed by Dunn’s multiple comparison test). Similar results were observed after the 20-min bath application of H_2_O_2_. No significant difference was observed in the membrane capacitance, peak potential, half width of the AP, or input resistance in response to H_2_O_2_ perfusion (Fig. S2C). Collectively, these data strongly indicate that ROS generated by NOX2 can rapidly induce mechanical allodynia and increase the excitability of DRG neurons.

### Pretreatment with the NOX2-blocking peptide gp91-tat inhibits SNI-induced upregulation of PKCε

**Fig. 4:**
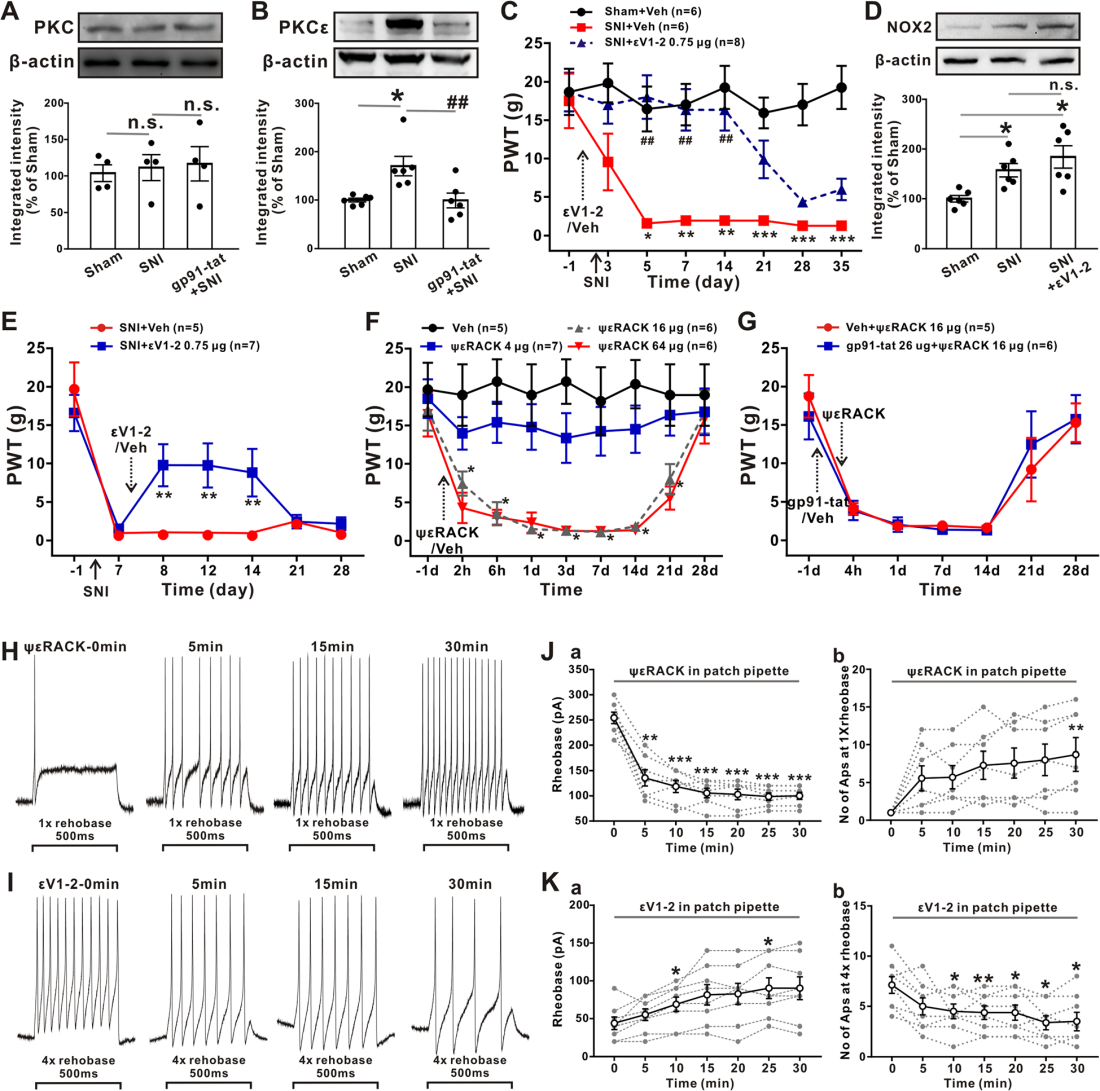
Pretreatment with the NOX2-blocking peptide gp91-tat inhibits the upregulation of PKCε. **a**, **b** Pretreatment with the gp91-tat inhibited the upregulated of PKCε (B) but not PKC (**a**) induced by SNI in L4-L6 DRGs (*n* = 4–5/group). One-way ANOVA followed by Tukey’s test. *p < 0.05 versus sham group; ##*p* < 0.01 versus the SNI group. **c** Local application of the PKCε-blocking peptide εV1-2 to L4-L6 DRGs before SNI reversed mechanical allodynia (*n* = 6–8/group). Kruskal-Wallis test followed by Dunn’s multiple comparison test. *p < 0.05, **p < 0.01, ***p < 0.001 versus the sham group; ##*p* < 0.01 versus SNI group. **d** Pretreatment with εV1-2 inhibited the SNI-induced the upregulated of NOX2 in L4–L6 DRGs (*n* = 6/group). One-way ANOVA followed by Dunnett’s T3 multiple comparisons test *p < 0.05 versus the sham group. **e** εV1-2 reversed SNI-induced mechanical allodynia at 1, 5, and 7 days after drug application (*n* = 5–7/group). Mann-Whitney *U* test. **p < 0.01 versus the vehicle group. **f** Local application of ψεRACK decreased the 50% PWT in a dose-dependent manner (*n* = 5–7/group). Mann-Whitney *U* test. *p < 0.05 versus the vehicle group. **g** Pretreatment with gp91-tat failed to affect ψεRACK-induced mechanical allodynia (*n* = 5–6/group). Mann-Whitney *U* test. **h** Representative traces of DRG neurons elicited by the 1× rheobase from before and at 5 min, 15 min, and 30 min after the application of ψεRACK (20 μM). **j**(a) Summary rheobase data (*n* = 7). One-way ANOVA followed by Tukey’s test. **p < 0.01, ***p < 0.001 versus the vehicle group. **j**(b) Number of APs (*n* = 7), Friedman test followed by Dunn’s multiple comparison test. **p < 0.01, ***p < 0.001 versus the vehicle group. **i** Representative traces of DRG neurons elicited by the 4× rheobase from before and after the application of εV1-2 (10 μM). **k** Summary rheobase data and the number of APs (*n* = 8). One-way ANOVA followed by Tukey’s test. *p < 0.05, **p < 0.01 versus the corresponding vehicle group

The interaction between oxidative stress and PKC is critically important in chronic pain and can amplify the development of central sensitization [28]. We hypothesized that NOX2 and PKC in DRGs interact with each other to impact neuropathic pain. To confirm this hypothesis, we investigated the effect of SNI-induced neuropathic pain on PKC expression in L4–L6 DRGs. However, Western blotting did not show any profound alterations in the protein expression of PKC after SNI (Fig. 4a). Of note, we observed that PKCε was significantly upregulated at 7 days after SNI (Fig. 4b), and this effect was inhibited by pretreatment with the NOX2-blocking peptide gp91-tat 2 h before SNI (from 170.21 ± 20.20% to 99.35 ± 14.92%, *p* 0.05, one-way ANOVA followed by Tukey’s test).

Next, to elucidate the up- and downstream relationship between these factors, we applied εV1-2, a PKCε-specific inhibitor, to L4–L6 DRGs 2 h before SNI. Similar to what was observed in gp91-tat-pretreated SNI rats, blocking PKCε significantly attenuated SNI-induced mechanical allodynia, which persisted to day 14 after SNI (Fig. 4c, at day 14 after SNI operation, 16.34 ± 5.08 g, *p* < 0.01 compared with the vehicle-treated SNI group, Kruskal-Wallis test followed by Dunn’s multiple comparison test). In parallel, we tested the effect of εV1-2 on NOX2 expression. We found that the SNI-induced increase in NOX2 protein expression in DRGs was not affected by pretreatment with εV1-2 (Fig. 4d, from 157.40 ± 12.51% in the SNI group to 184.18 ± 20.84% in the εV1-2-pretreated group, *p* > 0.05, one-way ANOVA followed by Dunnett's T3 multiple comparisons test), suggesting that PKCε activation is preceded by NOX2 activation. Consistently, the local application of εV1-2 at day 7 after SNI partially decreased the SNI-induced 50% PWT, which persisted for 7 days (Fig. 4e, 9.79 ± 5.61 g at day 8 after SNI, *p* < 0.01, 8.81 ± 6.71 g at day 14 after SNI, *p* < 0.01, Mann-Whitney *U* test). However, local application of the specific PKCε activator ψεRACK to L4–L6 DRGs rapidly induced mechanical allodynia in naïve rats in a dose-dependent manner. As shown in Fig. 4f, 16 μg and 64 μg of ψεRACK reduced the 50% PWT at 2 h, and the effect persisted for 21 days, whereas 4 μg of ψεRACK had no effect on the PWT. To further verify that the activation of PKCε was followed by NOX2 activation, we next examined whether pharmacological blockade of NOX2 could inhibit PKCε-induced mechanical allodynia. However, pretreatment with gp91-tat 2 h before ψεRACK injection did not have a significant effect on the 50% PWT decrease (Fig. 4g).

### Intracellular application of ψεRACK and εV1-2 rapidly modulates the excitability of DRG neurons

The rapid effect of the PKCε activator ψεRACK on mechanical allodynia suggests that the excitability of DRG neurons can be modulated by PKCε activation or inhibition. To explore this hypothesis, ψεRACK (20 μM) or εV1-2 (10 μM) was added to the intracellular solutions, and whole-cell patch clamp recordings were performed on DRG neurons from naïve rats or rats at 7 days after SNI. As shown in Fig. 4h and i, ψεRACK decreased the rheobase at 5–30 min and increased the number of APs in response to the 1× rheobase at 30 min in naïve rats. In contrast, εV1-2 markedly increased the rheobase at 10 and 25 min and decreased the number of APs elicited by the 4× rheobase at 10, 15, 20, 25, and 30 min (Fig. 4j and k). Neither ψεRACK nor εV1-2 had any effect on membrane capacitances, peak amplitudes of AP, half-widths of AP, or input resistance (Fig. S3).

### Pretreatment with gp91-tat prevents the SNI-induced plasma membrane translocation of PKCε in DRG neurons

**Fig. 5:**
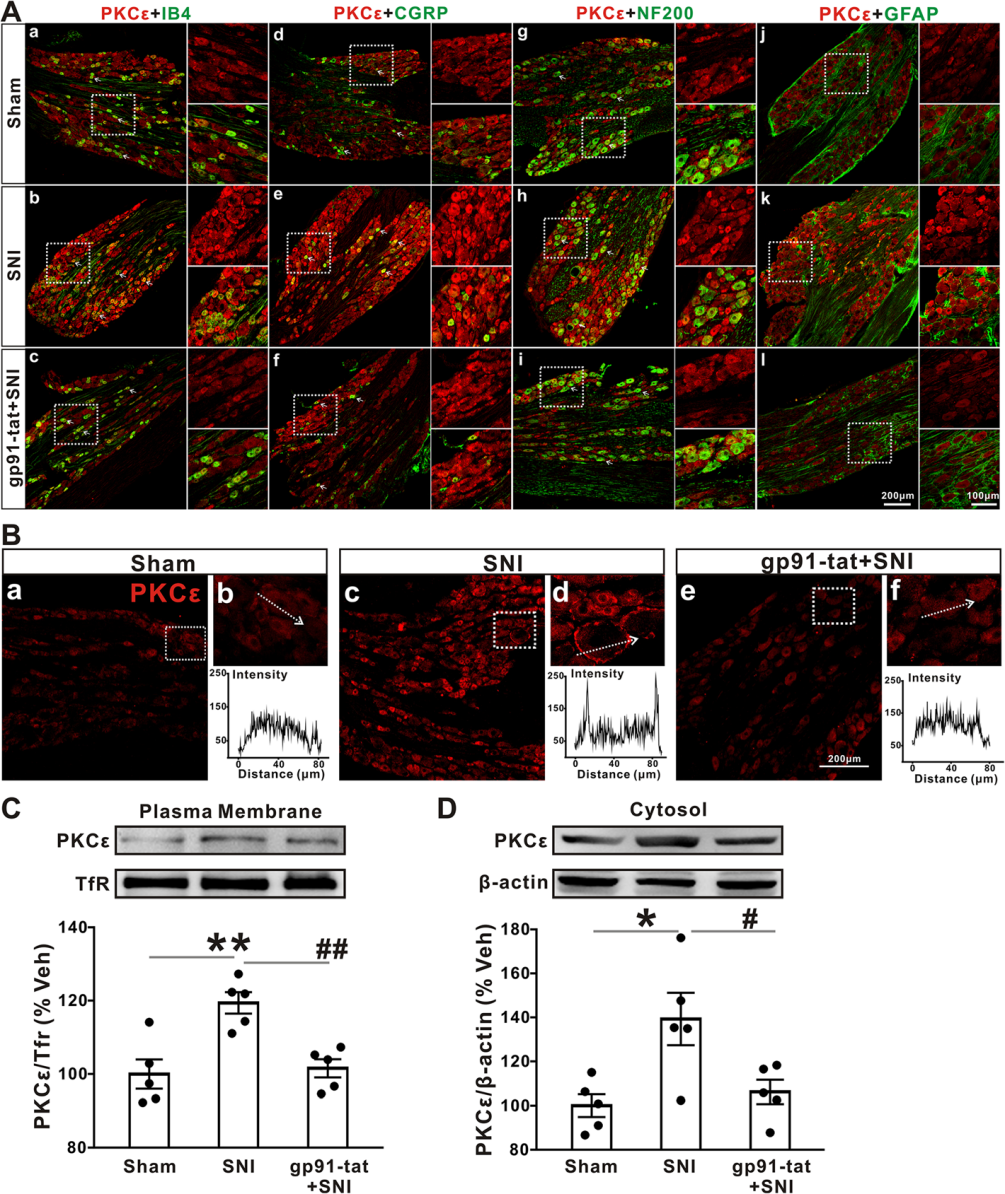
Pretreatment with gp91-tat prevents SNI-induced plasma membrane translocation of PKCε in DRG neurons. **a** Representative double-immunofluorescence staining showing the colocalization of PKCε with IB4 (**a**(a–c)), CGRP (**a**(d–f)), and NF-200 (**a**(g–i)) but not GFAP (**a**(j–l)) in the sham, SNI, and SNI pretreated with gp91-tat groups (*n* = 3/group). **b** Representative staining showing the plasma membrane translocation of PKCε in the SNI group (**b**(c, d)) but not in the sham (**b**(a, b)) and gp91-tat-pretreated groups (**b**(e, f)) presented as the fluorescence intensity of PKCε in the cell. **c**, **d** PKCε expression in both the plasma membrane (**c**) and cytosolic fractions (**d**) of DRGs from the sham, SNI, and gp91-tat-pretreated SNI groups was determined by Western blotting (*n* = 5/group). One-way ANOVA followed by Tukey’s test. *p < 0.05, **p < 0.01 versus the sham group; #*p* < 0.05, ##*p* < 0.01 versus the SNI group

Double immunofluorescence staining showed that PKCε colocalized with IB4-, CGRP-, and NF-200-labeled neurons (Fig. 5a(a–c), (d–f), and (g–i), respectively) but not with GFAP-labeled cells (Fig. 5a(j–l)) in the L4-L6 DRGs of sham rats, rats at 7 days after SNI, and gp91-tat-pretreated rats at 7 days after SNI. Interestingly, we found that SNI caused the translocation of PKCε from the cytosol to the plasma membrane in L4–L6 DRG neurons, which was significantly attenuated by gp91-tat pretreatment (Fig. 5b(a–f)). To further confirm the membrane translocation of PKCε, we next performed WB on the subcellular fraction and verified that PKCε protein expression was upregulated in both the plasma membrane and cytosol at day 7 after SNI (119.38 ± 2.93% and 139.28 ± 11.89% of sham control, *p* < 0.01 and *p* < 0.05, respectively, one-way ANOVA followed by Tukey’s test, Fig. 5c, d), and this effect was significantly decreased by gp91-tat pretreatment (101.55 ± 2.50% and 106.19 ± 5.50%, *p* < 0.01 and *p* < 0.05, respectively, one-way ANOVA followed by Tukey’s test).

### H_2_O_2_ causes the translocation of PKCε to the plasma membrane

**Fig. 6:**
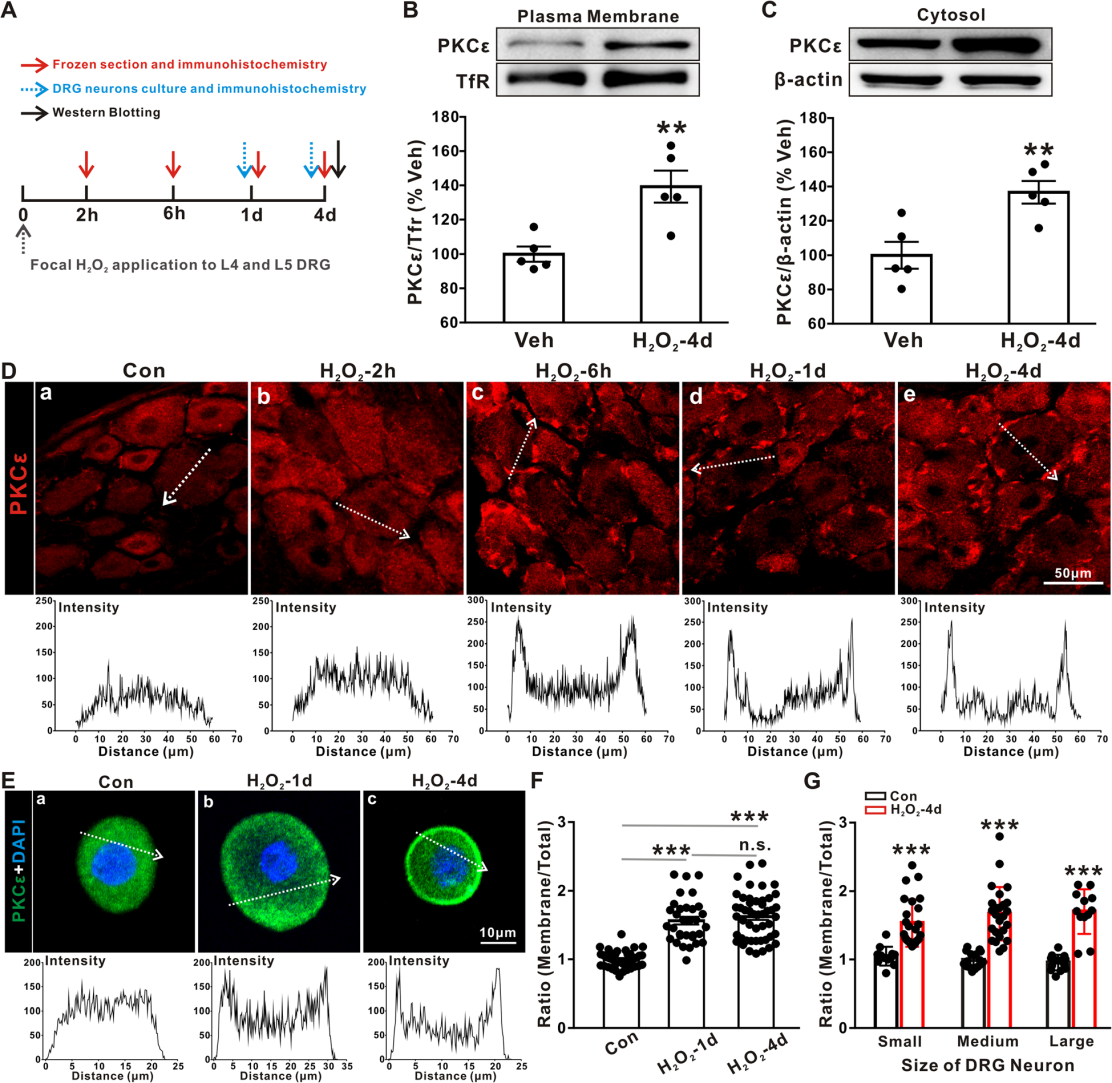
H_2_O_2_ induces the plasma membrane translocation of PKCε. **a** Experimental diagram showing the timeline of H_2_O_2_ application, immunohistochemistry, and Western blot analysis. **b**, **c** Western blot showing that PKCε protein expression was increased in both plasma membrane (**b**) and cytosol (**c**) of DRGs after the local application of H_2_O_2_ (*n* = 5/group). Two-tailed unpaired Student’s *t* test. **p < 0.01 versus the vehicle group. **d** Representative staining illustrating the plasma membrane translocation of PKCε in DRG sections after 1 and 4 days of local H_2_O_2_ administration. The fluorescence intensity of PKCε in the cell (indicated by the arrow) is illustrated. **e** Representative staining showing that PKCε translocated to the plasma membrane in cultured L4-L6 DRG neurons after 1 and 4 days of H_2_O_2_ treatment. The fluorescence intensity of PKCε in the cell (indicated by the arrow) is illustrated. **f** Ratios of the PKCε fluorescence intensity in the membrane and total soma of control cells and at 1 day and 4 days after drug application (*n* = 32–48/group). One-way ANOVA followed by Dunnett's T3 multiple comparisons test. ***p < 0.001 versus the control group. **g** Quantitative measurements showing the fluorescence intensity ratios of membrane and total soma in small, medium, and large DRGs (*n* = 12–25/group). One-way ANOVA followed by Dunnett's T3 multiple comparisons test. ***p < 0.001 versus the control group

Next, we examined whether the expression and subcellular distribution of PKCε could be regulated by oxidative stress through the administration of H_2_O_2_ (Fig. 6a). As shown in Fig. 6b and c, at day 4 after H_2_O_2_ treatment, the protein expression of PKCε was significantly upregulated to 139.28 ± 9.34% and 136.65 ± 6.64% in the plasma membrane and cytosol of cultured DRGs, respectively, compared to those of vehicle-treated rats (100.00 ± 4.43% in the membrane; 100.00 ± 7.87% in the cytosol; *p* < 0.01, two-tailed unpaired Student’s *t* test).

To further confirm the effect of H_2_O_2_ on the subcellular distribution of PKCε, we performed immunostaining of PKCε in frozen sections of DRGs obtained from control and H_2_O_2_-treated rats. Interestingly, H_2_O_2_ treatment induced membrane translocation of PKCε in DRGs, which began at 1 h and persisted for at least 4 days after H_2_O_2_ treatment (Fig. 6d). Furthermore, we investigated the translocation of PKCε in cultured DRG neurons. As shown in Fig. 6e and f, at day 1 after treatment with H_2_O_2_, the average distribution ratio of the membrane to soma was higher than that of the control rats and was maintained at a higher level at day 4 in H_2_O_2_-treated DRGs (from 0.99 ± 0.02 in the control group to 1.62 ± 0.05 in the 4-day H_2_O_2_-treated group, *p* < 0.001, one-way ANOVA followed by Dunnett’s T3 multiple comparisons test). Moreover, the translocation of PKCε was observed in small (< 25 μm), medium (25–35 μm), and large (> 35 μm) DRG neurons (Fig. 6g). Taken together, these results indicated that the direct application of H_2_O_2_ may induce the plasma membrane translocation of PKCε in DRG neurons.

### Oxidative stress involvement in pain behavior is mediated by the plasma membrane translocation of PKCε

**Fig. 7:**
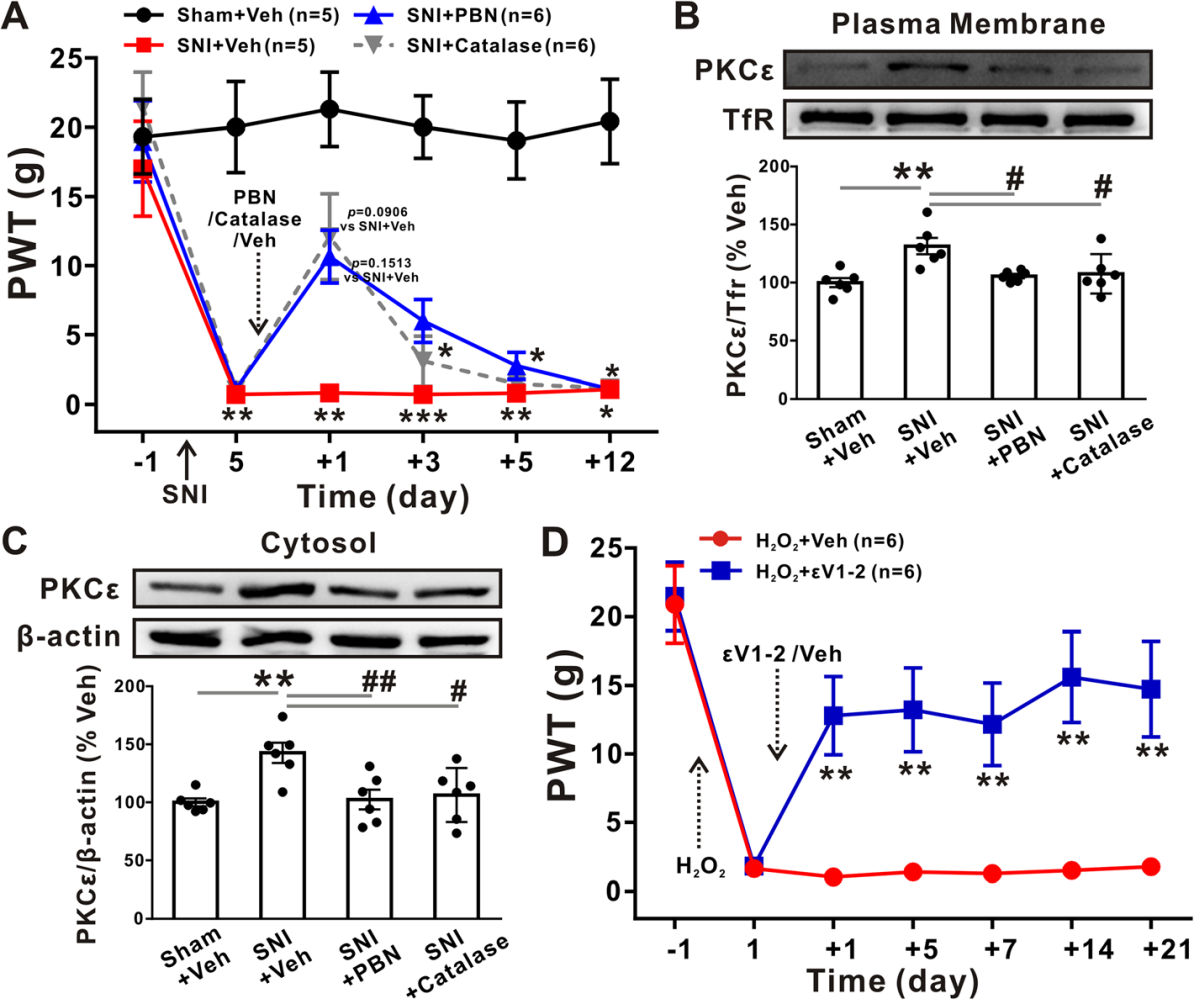
Oxidative stress induces mechanical allodynia via the plasma membrane translocation of PKCε. **a** Effects of PBN and catalase on the 50% PWT after drug application. The dotted arrow indicates the time of PBN, catalase, or vehicle application (*n* = 5–6/group). Kruskal-Wallis test followed by Dunn’s multiple comparison test. *p < 0.05, **p < 0.01, ***p < 0.001 versus the sham group. **b**, **c** Treatment with PBN or catalase 1 day after SNI inhibited the upregulation of PKCε protein expression in both the plasma membrane (**b**) and cytosol (**c**) of DRGs (*n* = 6/group). One-way ANOVA followed by Tukey’s test. **p < 0.01 versus the vehicle-treated sham group; #*p* < 0.05, ##*p* < 0.01 versus the vehicle-treated SNI group. **d** εV1-2 reversed H_2_O_2_-induced mechanical allodynia (*n* = 6/group). Mann-Whitney *U* test. **p < 0.01 versus the vehicle-treated H_2_O_2_ group

Treatment with the ROS scavenger PBN (100 μg in 2 μl per DRG) and the specific H_2_O_2_ catalyst catalase [31] (500 units in 2 μl per DRG) decreased SNI-induced PWT after 1 day (from 0.71 ± 0.07 g in the vehicle-treated SNI group to 10.66 ± 3.72 g in the PBN-treated SNI group, *p* = 0.1513, and 12.09 ± 5.99 in the catalase-treated SNI group, *p* = 0.0906, Kruskal-Wallis test followed by Dunn’s multiple comparison test, Fig. 7a). The Western blot analysis showed that PBN and catalase treatment 1 day after SNI abrogated the SNI-induced increase in PKCε protein in both the plasma membrane and cytosol (Fig. 7b and c). Moreover, treatment with εV1-2 after H_2_O_2_ reversed the decrease in PWT from 1- (1.03 ± 0.09 in the H_2_O_2_ treatment with vehicle group compared with 12.79 ± 5.99 in the H_2_O_2_ treatment with εV1-2 group, *p* < 0.01, Mann-Whitney *U* test, Fig. 7d) until the end of the pain behavior test. Taken together, these data suggest that the activation of superoxide and H_2_O_2_ is required for the upregulation of PKCε protein in both the plasma membrane and cytosol, which ultimately leads to SNI-induced neuropathic pain.

## Discussion

**Fig. 8:**
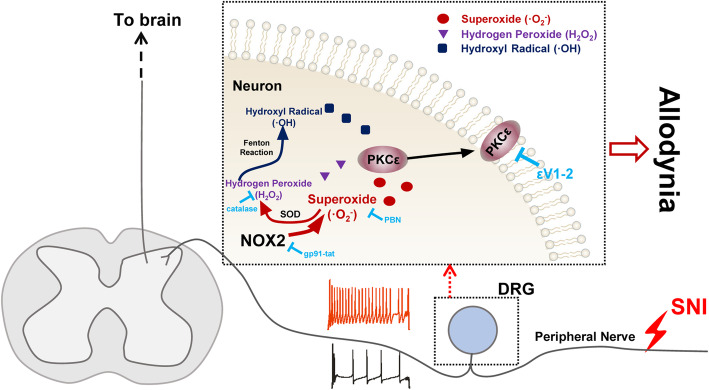
The mechanism of NOX2-ROS-PKCε signaling pathway contributes to peripheral sensitization and mechanical allodynia

In this study, we proposed a novel mechanism underlying the generation of neuropathic pain. SNI-induced upregulation of NOX2 generates ·O_2_^−^, H_2_O_2_, and ·OH, which in turn promote the plasma membrane translocation of PKCε in neurons. Inhibition of NOX2 and PKCε or treatment with ROS-scavenging agents can attenuate the hyperexcitability of DRG neurons and mechanical allodynia. The suppression of NOX2 by local pretreatment of DRGs with gp91-tat or NOX2-shRNA effectively reduced SNI-induced mechanical allodynia, which was also attenuated by PBN, catalase, and εV1-2 (Fig. 8). Moreover, the hyperexcitability of DRG neurons and increased levels of 8-OHG, ·OH, and PKCε after SNI were reversed by gp91-tat administration. We further showed that PKCε translocated from the cytosol to the plasma membrane in DRG neurons after SNI, and this effect was blocked by gp91-tat and mimicked by H_2_O_2_ administration in naïve animals. Finally, H_2_O_2_-induced mechanical allodynia was suppressed by εV1-2. Therefore, we concluded that PKCε membrane trafficking mediated by NOX2-induced oxidative stress after peripheral nerve injury promoted peripheral nociceptive sensitization.

### Oxidative stress is a key link between upstream NOX2 and downstream PKCε in DRGs

Accumulating evidence indicates that NOX2 plays an important role in neuropathic pain by altering the balance of pro- and anti-inflammatory cytokines, such as TNFα, IL-1β, and IL-10 [32, 33]. Our previous results showed that NOX2 was required for synaptic plasticity in the SDH and contributes to HFS-induced mirror-image pain [11]. In the present study, we showed that NOX2 was upregulated in DRGs after SNI. This upregulation was suppressed by the gp91-tat peptide or shRNA treatment before SNI, which attenuated mechanical allodynia, suggesting a critical role for NOX2 in initiating neuropathic pain. It reported that gp91-tat prevented phosphorylation of p47PHOX last to 7 day, treated with gp91-tat had a significant behavior improvement at 14- and 28-day time points after spinal cord injury (SCI), suggesting a long-term effect of gp91-tat in SCI mice [34]. In contrast, gp91-tat failed to alleviate pain if it was administered after SNI; the behavior test also found that application of PBN or catalase had a limited effect on mechanical allodynia induced by SNI. It implies that the activation of NOX2-ROS in DRGs by peripheral nerve injury triggered multiple downstream mechanisms. These results indicate that NOX2 is a good target for neuropathic pain prevention. It has been reported that NOX2 in DRG macrophages contributes to neuropathic pain by releasing proinflammatory cytokines to stimulate sensory neurons [12]. Additionally, it has been shown that NOX2 is expressed in the soma of trigeminal ganglion neurons [4]. In this study, we found that in rat DRGs, NOX2 was mainly expressed in neurons, with less expression in macrophages and no expression in satellite glial cells. These results indicated that NOX2 induced oxidative stress regulated neuro-excitability in DRG through direct neuronal interactions and the indirectly interactions of macrophage and neuron. As expected, the blockade of NOX2 deceased the hyperexcitability of DRG neurons. Therefore, we concluded that NOX2 promoted neuropathic pain by modulating neural excitability. However, we could not exclude the role of the macrophage-neuronal NOX2 signaling in SNI.

NOX2 is an important source of both extracellular and intracellular ROS [35]. Increase ROS induce pain by reducing GABA inhibitory transmission in substantia gelatinosa neurons of the SDH [2]. In DRG neurons, the sensitization of transient receptor potential ankyrin 1 to H_2_O_2_ leads to cold sensitivity [36]. Our data showed that SNI increased 8-OHG and ·OH levels in DRGs, and this effect was inhibited by the NOX2-blocking peptide. Nonnuclear 8-OHG immunostaining, which indicates the exposure of mitochondrial DNA to excessive oxidative stress [30], was also inhibited by pretreatment with gp91-tat before SNI. Moreover, we found that the application of H_2_O_2_ to DRGs dose-dependently induced mechanical allodynia and hyperexcitability in DRG neurons. These results suggest that ROS and H_2_O_2_ activation is an important mediator of NOX2 signaling in DRGs in the context of neuropathic pain.

It was reported that NOX2-derived ROS in the spinal cord could modulate PKC activity through sigma-1 receptors [18]. In addition, PKC is a regulator of NOX activity, as it induces the phosphorylation of NOX subunits such as gp91phox [37]. In this study, we found that gp91-tat inhibited the upregulation of PKCε but not PKC in DRGs after SNI. As PKCε is a key mediator of pain in DRGs [38], we next focused on the interaction of NOX2 and PKCε in this study. Our data showed that the PKCε inhibitor εV1-2 failed to affect the protein level of NOX2 and that mechanical allodynia induced by the PKCε activator ψεRACK was not attenuated by gp91-tat. Furthermore, εV1-2 treatment was sufficient to alleviate SNI-induced mechanical allodynia. These findings indicate that the upregulation of NOX2 after SNI is an important upstream activator of PKCε that ultimately contributes to the development of neuropathic pain in DRGs. ROS may be a key link between upstream NOX2 and downstream PKCε.

### Oxidative stress induced by NOX2 enhances the translocation of PKCam PKCεportant upstream activator of bitor gp91

Rather than the upregulation of PKCε, the translocation of PKC from the cytosolic (soluble) fraction to the plasma membrane is a signal of its activation [39]. The translocation of PKC isoforms has been reported in many neural diseases, including postmortem brain samples of patients with bipolar disorder [40] and the DRGs of rats with paclitaxel-induced peripheral neuropathy [16]. Consistently, we found plasma membrane translocation of PKCε in DRG neurons and upregulated protein expression in the plasma membrane after SNI. However, the functions of PKCε in the plasma membrane of DRG neurons are still unknown. Wu et al. showed that ψεRACK enhanced the Nav1.8 current and induced mechanical hyperalgesia [13]. In contrast, PKCε-I, a selective PKCε-anchoring inhibitor, prevented the reduction in the peak Na^+^ current in the mouse hippocampus [41]. In addition, TRPV1 currents in DRG neurons could also be reduced by a PKCε translocation inhibitor (PKCε TIP) in the recording pipette or pretreatment with the PKC inhibitor BIM [17]. Furthermore, it showed that motor protein kinesins, such as Kinesin superfamily proteins (KIFs), played an important role in neuronal cargo trafficking [42]. The KIF3 could motor transports atypical protein kinase C (aPKC) to the tip of nascent axons [43]. The effect of motor protein KIFs might be a trafficking mechanism that underling the plasma membrane translocation of PKCε induced by ROS. These reports raise the possibility that the plasma membrane translocation of PKCε allows it to be anchored to specific subcellular sites, which enables inhibitors and activators to be more effective. This possibility was further validated in our study by the intracellular application of a PKCε inhibitor or activator. As expected, εV1-2 decreased the excitability of DRG neurons in SNI rats, whereas ψεRACK rapidly increased the excitability of DRG neurons in naïve rats within minutes. However, whether the mechanism underlying the rapid regulation of DRG excitability by PKCε translocation occurs via interactions with other channels, such as sodium channels or TRPV1, in the plasma membrane needs to be further studied.

PKCε translocation in DRG neurons is affected by a series of factors. It was reported that ROS derived from NOX1 accelerated PKCε translocation by modulating the redox state of cysteine residues in DRG neurons and enhanced inflammatory pain [5]. Moreover, TGF-β which could be activated by redox imbalance [44] also induced the translocation of PKCε in DRG neurons [17]. In addition, bradykinin (BK), a peptide that enhances the membrane ionic current in nociceptive neurons, induces PKCε translocation to the cell membrane when it is activated by heat stimulation [45]. Furthermore, BK, activin A, and phorbol-12-myristate-13-acetate (a potent PKC activator) were reported to be involved in the translocation of PKCε, followed by TRPV1 channel sensitization and thermal hyperalgesia generation [46]. Notably, BK may induce NOX to generate ROS [47], and activin A is also related to ROS production [48]. These reports suggest that ROS may be an important source of PKCε cargo to the plasma membrane. In this study, we demonstrated that the plasma membrane translocation of PKCε and the upregulation of PKCε protein levels in both the plasma membrane and cytosol after SNI were blocked by gp91-tat pretreatment in DRGs. Similar results were observed after the local application of H_2_O_2_ to DRGs. To determine the unique roles of ROS and H_2_O_2_, PBN and catalase were used to scavenge ROS and catalyze H_2_O_2_, respectively. We found that both treatments decreased the upregulation of PKCε protein expression in the plasma membrane and cytosol. Most importantly, εV1-2 attenuated H_2_O_2_-induced mechanical allodynia. Collectively, the generation of ROS and H_2_O_2_ by NOX2 after SNI is a key step in the activation of PKCε, which ultimately leads to neuropathic pain.

## Conclusion

Our study demonstrated that NOX2-induced oxidative stress in DRG neurons is an important upstream factor of PKCε in the context of SNI conditions. In particular, oxidative stress induces DRG neuron hyperexcitability and the plasma membrane translocation of PKCε, ultimately contributing to peripheral sensitization and pain sensitivity in rats.

## Supplementary Information

**Additional file 1 Supplemental Figure 1.** The expression of NOX2 in L4-6 DRGs in the sham group. (A) Representative double-immunofluorescence staining showing the colocalization of NOX2 with IB4 (Aa), CGRP (Ab), and NF-200 (Ac) but not with CD11b (Ad) or GFAP (Ae). The percentages of each cell markers that expressed NOX2-IR in DRGs are shown (Af) in the sham group (n=6/group).

**Additional file 2 Supplemental Figure 2.** The effect of H_2_O_2_ on DRGs. (Aa-d) Double-immunofluorescence staining showing the colocalization of 8-OHG with IB4 (Aa), CGRP (Ab), and NF-200 (Ac) but not GFAP (Ad) (n=3/group). (B) Effects of H_2_O_2_-induced ·OH generation (n=5/group). Two-tailed unpaired Student’s *t* test. **p < 0.01 versus the vehicle group. (C) The protein levels of NOX2 in L4-L6 DRGs were not significantly different between the vehicle and H_2_O_2_ treatment groups (n=8/group). (D) Summary data showing the statistical comparisons of electrophysiological parameters before and after the application of H_2_O_2_ (n=6).

**Additional file 3 Supplemental Figure 3.** Statistical comparisons of electrophysiological parameters before and after the application of ψεRACK and εV1-2. Quantification of the membrane capacitances (Aa, Ba), peak amplitudes (Ab, Bb) and half-widths (Ac, Bc) of action potentials, as well as input resistance (Ad, Bd), before and after drug application (n=7-8/group).

## Data Availability

The authors should be contacted if any data or material is required to be provided.

## Abbreviations

NOX2: Nicotinamide adenine dinucleotide phosphate oxidase 2
ROS: Reactive oxygen species
PKC: Protein kinase C
DRG: Dorsal root ganglion
SNI: Spared nerve injury
PBN: Phenyl-*N*-tert-butylnitrone
O_2_^−^: Superoxide anion
OH: Hydroxyl radical
H_2_O_2_: Hydrogen peroxide
Aps: Action potentials
PWT: Paw withdrawal threshold
