# Supplementary figures and images for: Oxidative stress induced by NOX2 contributes to neuropathic pain via plasma membrane translocation of PKCε in rat dorsal root ganglion neurons
Source: J Neuroinflammation. 2021 May 6;18:106. doi: 10.1186/s12974-021-02155-6 (PMC8101139; doi:10.1186/s12974-021-02155-6)

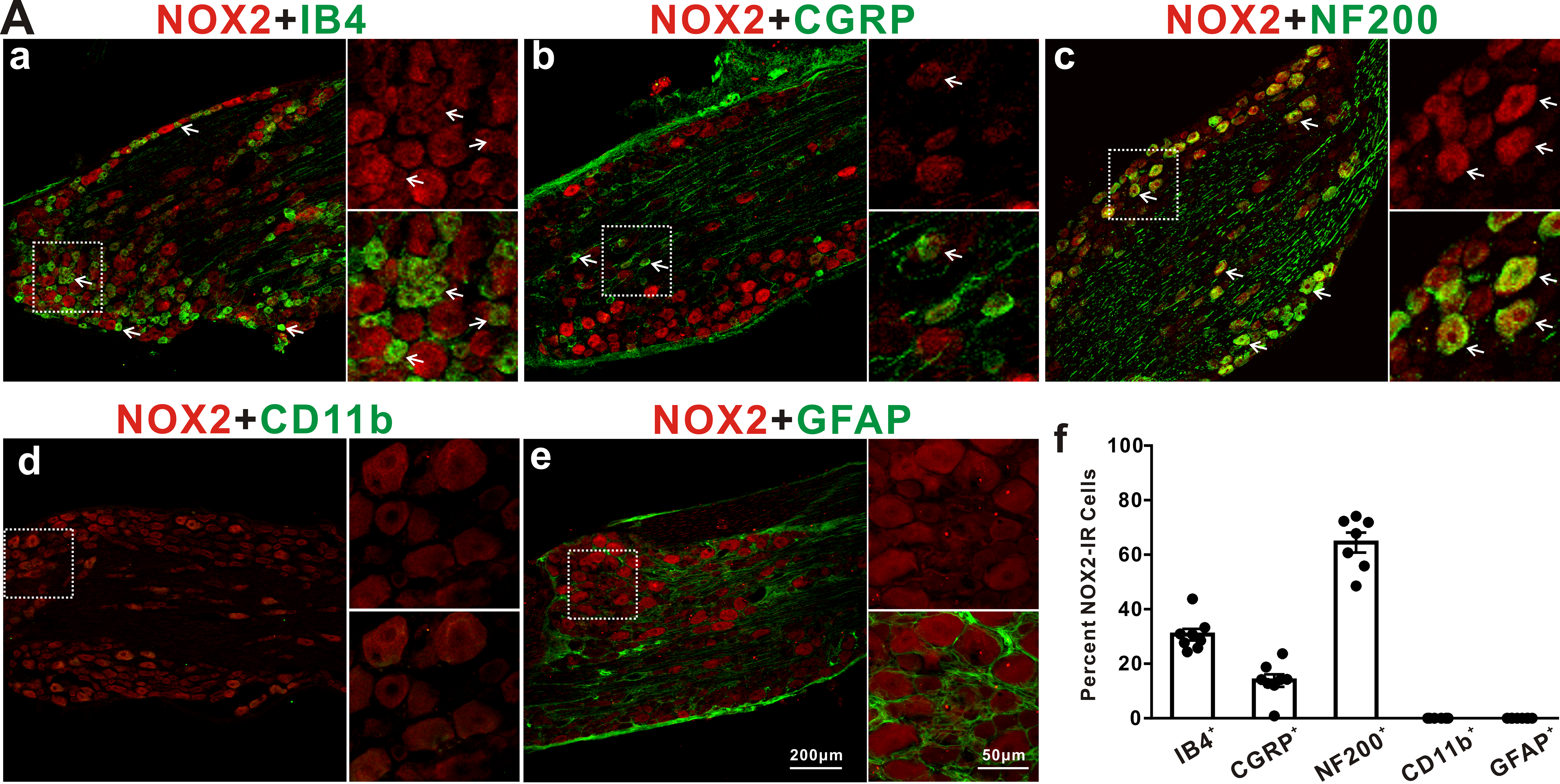

Supplement: Supplementary file 1 — Additional file 1 Supplemental Figure 1. The expression of NOX2 in L4-6 DRGs in the sham group. (A) Representative double-immunofluorescence staining showing the colocalization of NOX2 with IB4 (Aa), CGRP (Ab), and NF-200 (Ac) but not with CD11b (Ad) or GFAP (Ae). The percentages of each cell markers that expressed NOX2-IR in DRGs are shown (Af) in the sham group (n=6/group). [file 12974_2021_2155_MOESM1_ESM.tif]

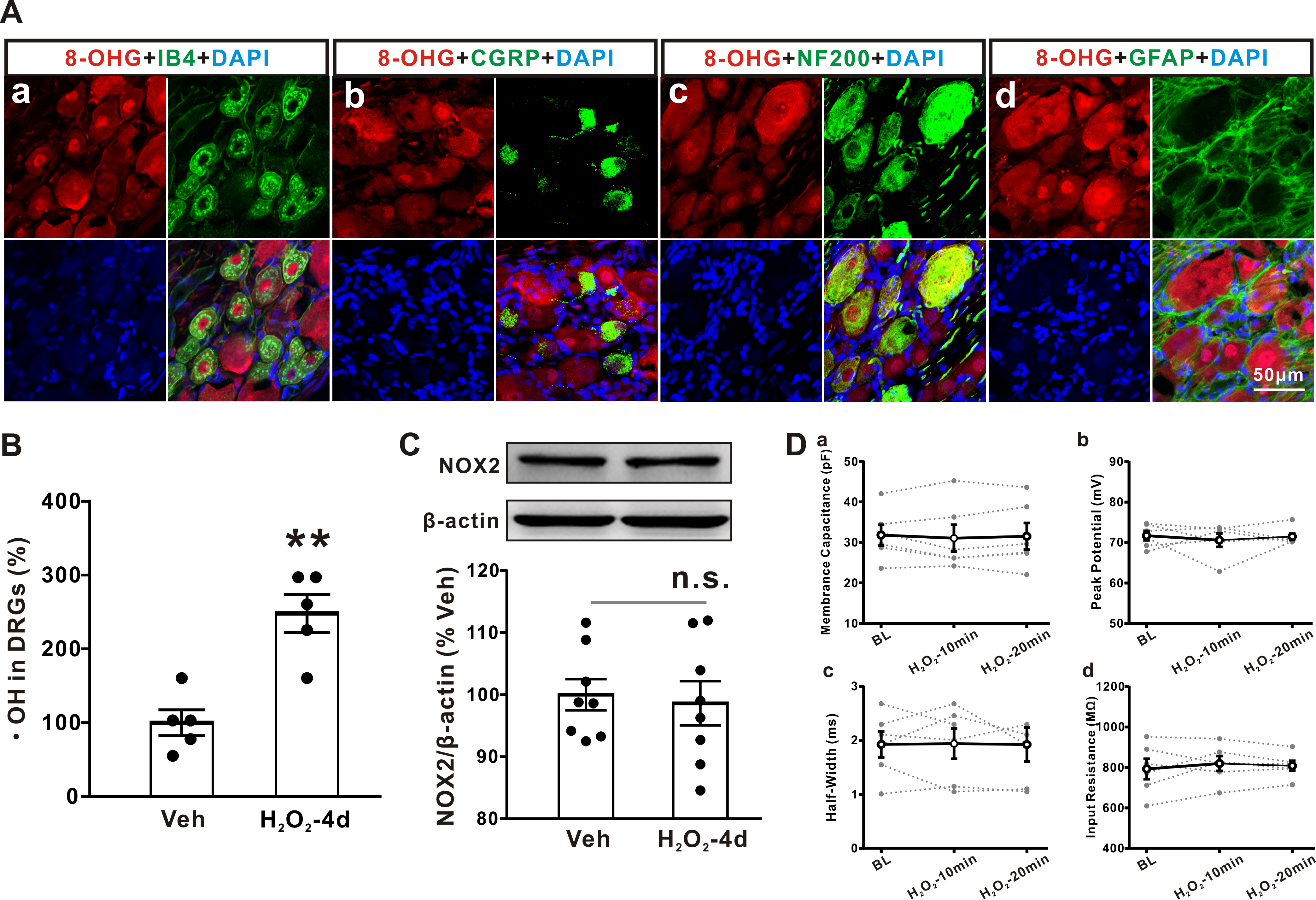

Supplement: Supplementary file 2 — Additional file 2 Supplemental Figure 2. The effect of H2O2 on DRGs. (Aa-d) Double-immunofluorescence staining showing the colocalization of 8-OHG with IB4 (Aa), CGRP (Ab), and NF-200 (Ac) but not GFAP (Ad) (n=3/group). (B) Effects of H2O2-induced ·OH generation (n=5/group). Two-tailed unpaired Student’s t test. **p < 0.01 versus the vehicle group. (C) The protein levels of NOX2 in L4-L6 DRGs were not significantly different between the vehicle and H2O2 treatment groups (n=8/group). (D) Summary data showing the statistical comparisons of electrophysiological parameters before and after the application of H2O2 (n=6). [file 12974_2021_2155_MOESM2_ESM.tif]

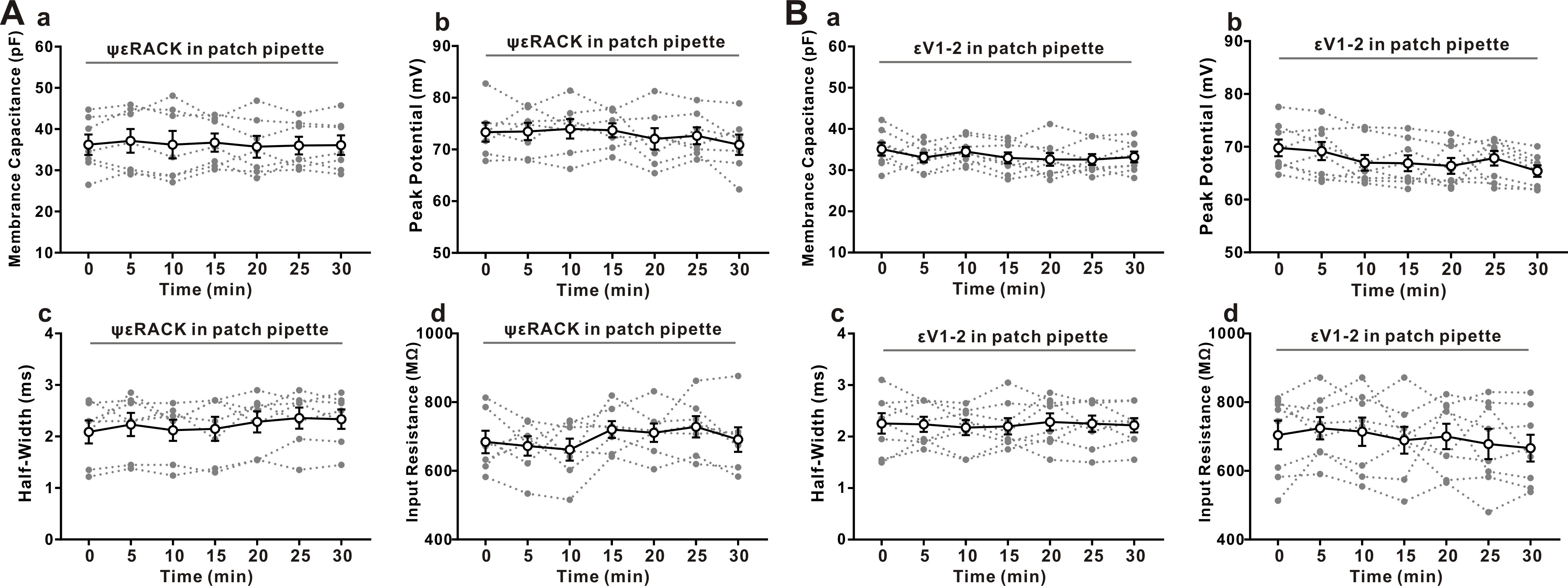

Supplement: Supplementary file 3 — Additional file 3 Supplemental Figure 3. Statistical comparisons of electrophysiological parameters before and after the application of ψεRACK and εV1-2. Quantification of the membrane capacitances (Aa, Ba), peak amplitudes (Ab, Bb) and half-widths (Ac, Bc) of action potentials, as well as input resistance (Ad, Bd), before and after drug application (n=7-8/group). [file 12974_2021_2155_MOESM3_ESM.tif]
